# Emergence of a Novel *Ehrlichia minasensis* Strain, Harboring the Major Immunogenic Glycoprotein trp36 with Unique Tandem Repeat and C-Terminal Region Sequences, in *Haemaphysalis hystricis* Ticks Removed from Free-Ranging Sheep in Hainan Province, China

**DOI:** 10.3390/microorganisms7090369

**Published:** 2019-09-19

**Authors:** Junjiao Li, Xinxin Liu, Jiaqi Mu, Xibing Yu, Yidong Fei, Jin Chang, Yuhai Bi, Yulong Zhou, Zhuang Ding, Renfu Yin

**Affiliations:** 1 Department of Veterinary Preventive Medicine, College of Veterinary Medicine, Jilin University, Xi’an Road 5333, Changchun 130062, China; 2 College of Food Science and Engineering, Jilin University, Xi’an Road 5333, Changchun 130062, China; 3 CAS Key Laboratory of Pathogenic Microbiology and Immunology, Institute of Microbiology, Chinese Academy of Sciences, Beijing 100101, China; 4 College of Animal Science and Veterinary Medicine, Heilongjiang Bayi Agricultural University, Daqing 163319, China

**Keywords:** *Ehrlichia minasensis*, *Haemaphysalis hystricis* tick, free-ranging sheep, South China, trp36

## Abstract

*Ehrlichia minasensis*, a recently described *Ehrlichia* species that is the most closely related to, but clearly distinct from, *Ehrlichia canis*, has been circulating in not only bovines, cervids, and dogs but also several tick species from Canada, Brazil, France, Pakistan, Ethiopia, and Israel. However, there are no reports of *E. minasensis* in China. The purpose of this study was to explore whether *E. minasensis* is present naturally in ticks in China. Through PCR targeting of the genus-conserved *dsb* gene, *E. minasensis* DNA was detected in *Haemaphysalis hystricis* ticks removed from free-ranging sheep in Hainan Province, South China in 2017. The partial sequence of the *dsb*, *16S rRNA*, and *groEL* genes demonstrated that the Hainan strain shared 99% identity with the *dsb* gene of *E. minasensis* strain UFMG-EV (GenBank: JX629808), with the *16S rRNA* of *E. minasensis* isolate E-2650 (MH500005) and with the *groEL* gene of *E. minasensis* strain UFMG-EV (JX629806), respectively. Moreover, sequence analysis of the major immunogenic tandem repeat protein (trp36) revealed that the Hainan strain harbored a unique tandem repeat sequence (APEAAPVSAPEAAPVSAPVS) and a C-terminal region that differed from those of other known *E. minasensis* strains. Additionally, phylogenetic analysis based on the entire amino acid sequence of trp36 revealed that the Hainan strain was closely related to a recently described *E. minasensis* strain from Brazil, of which the sister clade contained different strains of *E. canis*. The discovery of this novel Hainan strain in *H. hystricis* ticks represents the first known natural presence of *E. minasensis* in South China, highlighting the need for its constant surveillance.

## 1. Introduction

Ehrlichiosis, which is caused by an obligate, intracellular, gram-negative, tick-borne alphaproteobacterium within the genus *Ehrlichia* (family *Anaplasmataceae*), is an emerging disease in humans, domestic animals, and mice worldwide [1]. The genus *Ehrlichia* consists of five well-described species: *Ehrlichia chaffeensis*, *Ehrlichia ewingii*, *Ehrlichia canis*, *Ehrlichia ruminantium*, and *Ehrlichia muris* [2]. *Ehrlichia minasensis*, a recently recognized *Ehrlichia* species [3], is closely related to the canine monocytic ehrlichiosis-causing pathogen *E. canis*, with phylogenetic analysis revealing that this new species evolved from highly variable strains of *E. canis* [4]. The geographic distribution of *E. minasensis* is not limited to Canada and Brazil, as was previously reported [5,6,7], since recent works have discovered this bacterium in Ethiopia [8], France [9], Israel [10], Pakistan [11], and South Africa [12].

*E. minasensis* can be propagated in canine macrophage-like cell lines (e.g., DH82) and *Ixodes scapularis* cell lines (e.g., IDE8) [5,6], and can cause clinical manifestations associated with ehrlichiosis in experimentally infected cattle [5]. *E. minasensis* has been circulating among not only naturally infected dairy cattle, mule deer, and dogs, but also various tick species, including *Hyalomma marginatum*, *Hyalomma anatolicum*, and *Rhipicephalus microplus* [9,11,13]. However, whereas other *Ehrlichia* species (including *E. chaffeensis*) and *Ehrlichia*-like organisms have been detected in *Haemaphysalis hystricis* (*H. hystricis*), *E. minasensis* has not been detected in this tick species so far [14,15]. The hard-bodied *H. hystricis* (also named east Asian mountain haemaphysalid), which is an obligate ectoparasite of mammals, is distributed in China, Japan, Vietnam, India, and Thailand (http://www.catalogueoflife.org/col/details/species/id/4f85d86075bf0ac2ba1e6b55d31d82be).

A very limited number of epidemiologic surveillances works on *E. minasensis* in ticks and domestic animals have been performed. This neglect of *E. minasensis* detection was likely because these agents were considered to have a negligible economic impact on the livestock industry. However, efforts to discover the molecular and antigenic diversity of *E. minasensis* will unquestionably contribute to the development of effective vaccines and reliable immunodiagnostics for this disease as well as to unveiling the microbial factors associated with its disease pathogenesis. Furthermore, very limited information is available on *E. minasensis* in China. Therefore, the purpose of this study was to determine whether *E. minasensis* could be detected in ticks removed from free-ranging sheep in Hainan Province, South China.

## 2. Materials and Methods

### 2.1. Tick Collection and DNA Extraction

In June 2017, 82 adult ticks were removed from free-ranging sheep (*n* = 16) bred on one farm located in Haikou, Hainan Province (longitude 110.53, latitude 19.81), South China. The ticks were collected according to standardized sampling procedures [16] and were stored at −80 °C until tested. Total DNA was extracted directly from pooled tick samples (5 ticks per pool, same tick species and same host) using the Wizard^®^ Genomic DNA Purification Kit (Promega, Shanghai, China) according to the manufacturer’s instructions. The collected ticks were identified to the species level by PCR amplification targeting the *16S rRNA* gene fragment and the cytochrome coxidase subunit 1 (*cox1*) gene [17,18] (primer sequences and PCR conditions as shown in Table 1).

### 2.2. PCR Amplification and DNA Sequencing of the dsb, 16S rRNA, groEL, and trp36 Genes of E. minasensis

The purified DNA was tested in four individual PCR amplifications using primers targeting a portion of the disulfide bond formation protein (*dsb*) gene, the *16S rRNA* gene, the heat shock protein (*groEL*) gene and the glycoprotein trp36 (*trp36*) gene. The reactions (20 µL) contained 2 µL of template DNA, 0.5 mM of each primer, and 10 µL of 2 × EasyTaq PCR SuperMix (TransGen, Beijing, China). Detailed information about the primers and PCR condition is shown in Table 1. The positive PCR product was subjected to DNA sequencing (ABI PRISM 377 DNA sequencer). The full sequence for both strands of each DNA template was determined to ensure maximum accuracy of the data.

### 2.3. DNA Sequence Analysis and Phylogenetic Analysis

All sequences obtained in this study were assembled and compared with sequences available in the GenBank database, using the BLAST algorithm (http://blast.ncbi.nlm.nih.gov/Blast.cgi). The nucleotide sequences were translated to their corresponding amino acid (aa) sequences using the EMBOSS Transeq tool (https://www.ebi.ac.uk/Tools/st/emboss_transeq). The nucleic acid and aa alignments were performed with the ClustalW multiple sequence alignment application that is included in the BioEdit software package. Phylogenetic analyses based on the partial coding sequence (CDS) of the *dsb*, 16S *rRNA* and groEL genes and the aa sequence of trp36 were conducted in MEGA X [20]. The evolutionary history was inferred by using the maximum-likelihood method based on the Tamura-Nei model (*dsb*, *16S rRNA and groEL* genes) and the Jones–Taylor–Thornton (JTT) matrix-based model (trp36), respectively. Initial tree(s) for the heuristic search were obtained automatically by applying neighbor-joining (NJ) and BioNJ algorithms to a matrix of pairwise distances estimated using the JTT model (trp36) and a maximum composite-likelihood approach (*dsb*, *16S rRNA* and *groEL* genes), and then selecting the topology with a superior log likelihood value. The tree is drawn to scale, with branch lengths measured in the number of substitutions per site. The analysis involved 19 (*dsb*), 18 (*16S rRNA*), and 12 (*groEL*) nucleotide sequences and 10 amino acid sequences (1D). All positions containing gaps and missing data were eliminated. In total, 223 (*dsb*), 279(*16S rRNA*), and 530 (*groEL*) positions were in the final dataset.

## 3. Results and Discussion

### 3.1. Identification of Tick Species

The nucleic acid sequences of the *16S rRNA* and *cox1* genes indicated that all ticks collected in this study were of the *H. hystricis* species.

### 3.2. Sequence Analysis of the dsb, 16S rRNA and groEL Genes of E. minasensis

**Figure 1:**
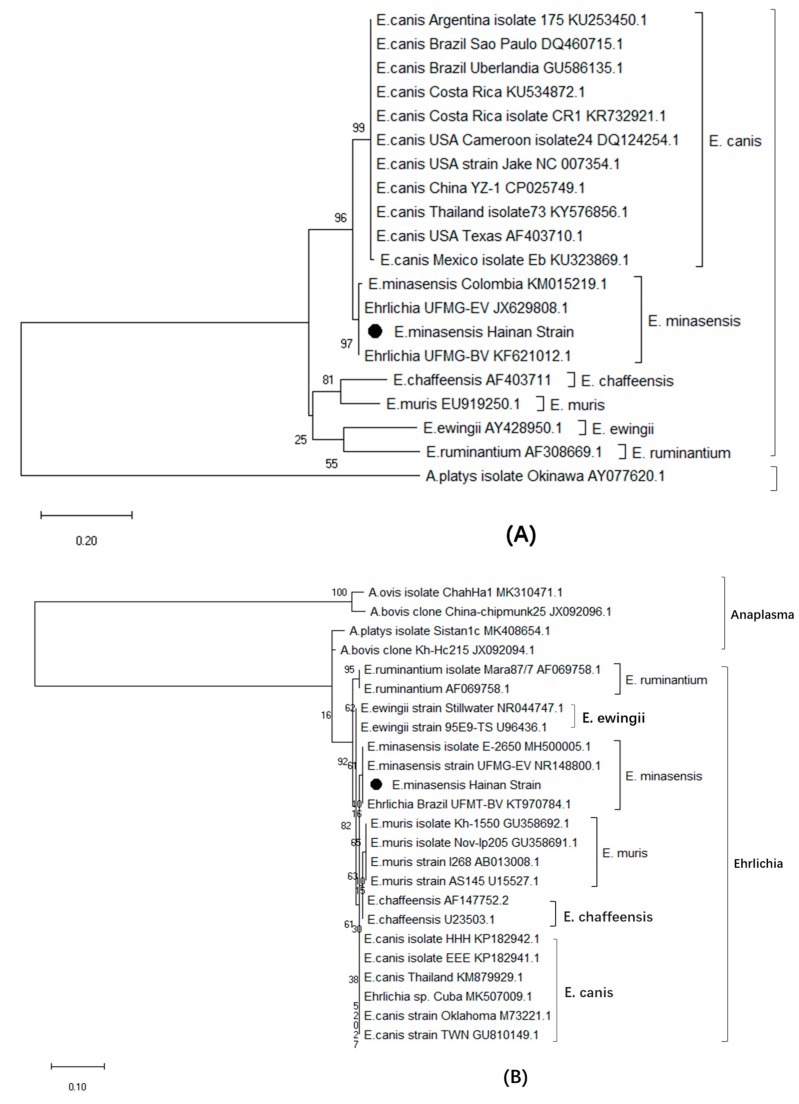

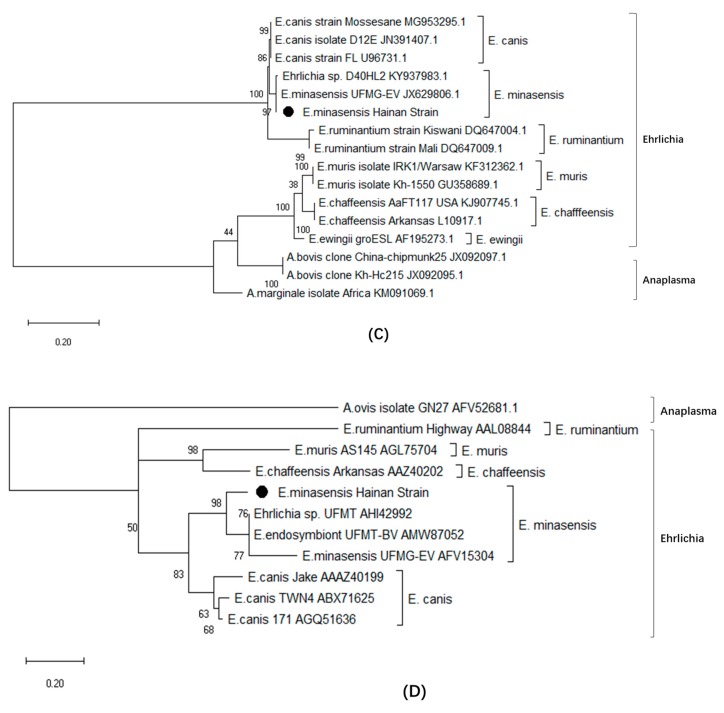
Phylogenetic tree based on the gene sequences of *dsb* (**A**), *16S rRNA* (**B**), *groEL* (**C**), and amino acid sequences of trp36 (**D**) from geographically dispersed *Ehrlichia minasensis* and *Ehrlichia canis* strains, as inferred by the maximum-likelihood method using other species of *Ehrlichia* as a genus outgroup and other strains of *Anaplasma* as a genuine outgroup. The tree with the highest log likelihood of –925.65, –449.26, –2007.15, and –2916.24 (**A**–**D**) are shown. The percentage of trees in which the associated taxa clustered together is shown next to the branches.

In this study, only one of the 16 sample pools (6.25%, 5 ticks from the same sheep) was PCR positive for the genus-conserved *dsb, 16S rRNA*, *groEL* genes of *E. minasensis* and *E. canis.* Upon comparison with sequences available from the GenBank database, the *dsb*, *16S rRNA*, and *groEL* genes of the *E. minasensis* Hainan strain identified in this study (GenBank MN463729) were found to have 99% partial CDS similarity to the *dsb* genes from *E. minasensis* strain UFMG-EV (JX629808; 363/365), isolate E-2650 (MH500007; 343/344), strain 1E (KM015219; 325/329) and to the *16S rRNA* of *E. minasensis* isolate E-2650 (MH500005; 342/345) as well as to the *groEL* gene of *E. minasensis* strain UFMG-EV (JX629806; 624/626), respectively. In addition, the phylogenetic tree based on the partial CDS of *dsb*, *16S rRNA*, and *groEL* genes revealed that the Hainan strain grouped together with *E. minasensis* into a clade of which the sister clade had different strains of *E. canis* (Figure 1A–C).

### 3.3. Sequence Analysis of the trp36 Gene

**Figure 2:**
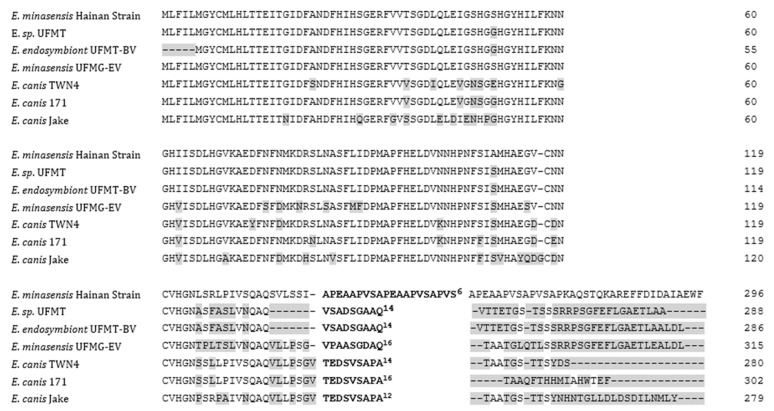
Comparison of the trp36 amino acid sequences from the Hainan strain and other strains of *Ehrlichia minasensis* (strains UFMT, UFMT-BV, and UFMG-EV) and strains of *Ehrlichia canis* (strains TWN4 and Jake). Amino acids highlighted in grey represent residues divergent from the *Ehrlichia minasensis* Hainan Strain sequence. The superscripted numbers correspond to the number of tandem repeats of the 9-20 residue unit.

The gene that encodes trp36 has been widely used as a target for molecular investigations of *E. canis* and *E. minasensis* and for distinguishing between the two species [4,5,21]; therefore, the complete *trp36* gene of the Hainan strain was amplified and sequenced. Sequencing of the PCR amplicon revealed that the *trp36* gene was 891 bp in size, encoding a predicted protein of 296 aa. According to the nucleotide sequence analysis, the *trp36* gene of the Hainan strain shared 97% identity with the *trp36* gene sequence of *E. minasensis* strain UFMT (KF870578; 395/406), 97% with the *trp36* gene of strain UFMT-BV (KT970785; 380/391), and 93% with the *trp36* gene of strain UFMG-EV (JX629809; 406/435), as well as 92% with the *trp36* genes of *E. canis* strain Bloemfontein (KC935387; 400/433), strain 222 (KC479021; 400/433), and strain 171 (KC479020; 400/433). The deduced aa sequence of the Hainan strain is shown in Figure 2.

### 3.4. Sequence Analysis of the N-Terminal Region and the Upstream Tandem Repeat Region of the trp36 Gene

The N-terminal region of trp36, which contained 141 aa, was 98% identical to that of *E. minasensis* strains UFMT (AHI42992; 123/126) and MFMT-BV (AMW87052; 118/121), whereas it shared 89% identity with that of *E. canis* strain 171 (AGQ51636; 115/129), 87% with that of strain TWN4 (ABX71625; 112/129), and 86% with that of strain Pocone C6 (KY522826; 360/419), when compared against aa sequences available from the GenBank database. In line with reported studies [5,6], the predicated aa sequence of the N-terminal region of trp36 from *E. minasensis* exhibited the highest identity (93–98%) with that from all reported *E. minasensis* strains. This minor diversity was also observed within *E. canis* (Figure 2), since there are no antibody epitopes in the N-terminal region and thus potentially less immune-driven adaptations [5,22]. Interestingly, the sequence upstream of the tandem repeat (TR) region (IVSQAQSVLSSI) of the Hainan strain was partially identical to that of *E. canis* strains from China and Thailand (ABS82573, ABU44524, ABV26011, CP025749, and MF771084: IVSQAQ***VL***L***P***S***G***), and completely different to that of *E. minasensis* strains from Brazil and Canada (AMW87052 and AHI42992: LVNQAQ; and AFV15304: LVNQAQVLLPSG) and of *E. canis* strains from Costa Rica, Peru, and Turkey (KU194227, MF095619, and MG905718: IVNQAQAILSSAT). However, the potential roles of the upstream TR region of the *trp36* genes from *E. minasensis* and *E. canis* are still unknown.

### 3.5. Sequence Analysis of the Tandem Repeat Region and C-Terminal Region of the trp36 Gene

The TR region of the *trp36* gene of the Hainan strain contained six TRs of 60 bp in length, each encoding 20 aa. The single TR had the sequence APEAAPVSAPEAAPVSAPVS and was completely different to the TR sequences reported for glycoprotein orthologs of trp36 from *E. canis* and *E. minasensis* (Figure 2). In addition, the C-terminal sequence of the gene from the Hainan strain was 105 bp in length, encoding 35 aa, which also differed from any previously reported *E. canis* and *E. minasensis* C-terminal sequences (Figure 2). Taken together, the results suggested that the TR aa sequence of trp36 was the most divergent region between this Hainan strain and *E. minasensis* and *E. canis*, suggesting that a recent TR diversification was likely driven by immune pressure, since the major antibody epitope is located in this region [5,6,22].

### 3.6. Phylogenetic Relationship Analysis Based on trp36

To further elucidate the genetic characteristics of this novel *E. minasensis* Hainan strain, a phylogenetic tree was generated on the basis of the entire aa sequence of trp36. According to the phylogenetic tree, which was built using the maximum-likelihood method based on the Jones–Taylor–Thornton (JTT) matrix-based model, this Hainan strain isolated from the *H. hystricis* tick clustered into the same clade as other *E. minasensis* strains from Brazil, of which the sister clade had different strains of *E. canis* [5,23]. The isolation of such highly similar clade strains from distinct animal and tick species in different regions of the Americas and Asia indicates that the *E. minasensis* strains could be intercontinental and interspecies transmitted by some specific way, such as via migrating birds [24,25]. However, no similar *E. minasensis* strain was detected in ticks from other regions of China, although 1060 adult ticks were collected from free-ranging livestock and pets in one province in East China (Zhejiang, *Haemaphysalis longicornis*, *n* = 18), two provinces in Northeast China (Jilin, *H. longicornis*, *n* = 282; and Heilongjiang, *Haemaphysalis japonica*, *n* = 349, *Dermacentor nuttalli*, *n* = 131, and *Ixodes persulcatus*, *n* = 73), and one province in North China (Inner Mongolia, *Dermacentor nuttalli*, *n* = 207) during the years 2016 to 2018. Therefore, our current data suggest that no *E. minasensis* strain was introduced into mainland China from Hainan Island of South China and that circulation of the strain was limited.

## 4. Conclusions

Our current data indicate that a novel *E. minasensis* strain, which harbors the major immunogenic glycoprotein trp36 with unique TR and C-terminal region sequences, existed in *H. hystricis* ticks removed from free-ranging sheep in South China but not in other regions of the country. However, further studies are needed to address the question of whether *H. hystricis* is a competent tick vector for this *E. minasensis* strain and whether this new bacterial strain is an emerging pathogen of sheep, goats or other ruminants, including dairy and beef cattle. In addition, our findings suggest a need for the constant epidemiologic surveillance for *E. minasensis* strains in domestic animals and wildlife in China in order to stay abreast of the potential introduction of novel variants from other ticks and hosts.

## Figures and Tables

**Table 1:** Primers used in this study.

Species	Target	Primer Name	Sequence	PCR Condition	Length	References
ticks	*16S rDNA*	16S+1 16S-1	CCGGTCTGAACTCAGATCAAG CTGCTCAATGATTTTTTAAATTGCTGTGG	95 °C 5 min, 35 × (95 °C 30 s, 57 °C 30 s, 72 °C 40 s), 72 °C 10 min	460 bp	[17]
	*cox1*	LCO1490 HCO2198	GGTCAACAAATCATAAAGATATTGG TAAACTTCAGGGTGACCAAAAAATCA	95 °C 5 min, 35 × (95 °C 30 s, 57 °C 30 s, 72 °C 40 s), 72 °C 10 min	650 bp	[18]
*Ehrlichia minasensis*	*dsb*	dsb-330 dsb-728	GATGATGTCTGAAGATATGAAACAAAT CTGCTCGTCTATTTTACTTCTTAAAGT	94 °C 5 min, 35 × (94 °C 30 s, 50.5 °C 60 s, 72 °C 60 s), 72 °C 10 min	400 bp	[19]
	*16S rRNA*	Ehr-16S-D Ehr-16S-R	GGTACCYACAGAAGAAGTCC TAGCACTCATCGTTTACAGC	94 °C 5 min, 35 × (94 °C 30 s, 54 °C 60 s, 72 °C 60 s), 72 °C 10 min	345 bp	[9]
	*groEL*	Ehr-groel-F Ehr-groel-R	GTTGAAAARACTGATGGTATGCA ACACGRTCTTTACGYTCYTTAAC	94 °C 5 min, 35 × (94 °C 30 s, 55 °C 60 s, 72 °C 60 s), 72 °C 10 min	590 bp	[9]
	*trp36*	TRP36-F2 TRP36-R1	TTTAAAACAAAATTAACACACTA AAGATTAACTTAATACTCAATATTACT	94 °C 5 min, 35 × (94 °C 30 s, 46 °C 60 s, 72 °C 60 s), 72 °C 10 min	800–1000 bp	[17]
